# Dependency of the Spindle Assembly Checkpoint on Cdk1 Renders the Anaphase Transition Irreversible

**DOI:** 10.1016/j.cub.2014.01.033

**Published:** 2014-03-17

**Authors:** Ahmed Rattani, P.K. Vinod, Jonathan Godwin, Kikuë Tachibana-Konwalski, Magda Wolna, Marcos Malumbres, Béla Novák, Kim Nasmyth

**Affiliations:** 1 Department of Biochemistry, University of Oxford, South Parks Road, Oxford OX1 3QU, UK; 2 Cell Division and Cancer Group, Spanish National Cancer Research Center (CNIO), 28029 Madrid, Spain

## Abstract

Activation of anaphase-promoting complex/cyclosome (APC/C^Cdc20^) by Cdc20 is delayed by the spindle assembly checkpoint (SAC). When all kinetochores come under tension, the SAC is turned off and APC/C^Cdc20^ degrades cyclin B and securin, which activates separase [1]. The latter then cleaves cohesin holding sister chromatids together [2]. Because cohesin cleavage also destroys the tension responsible for turning off the SAC, cells must possess a mechanism to prevent SAC reactivation during anaphase, which could be conferred by a dependence of the SAC on Cdk1 [3–5]. To test this, we analyzed mouse oocytes and embryos expressing nondegradable cyclin B together with a Cdk1-resistant form of separase. After biorientation and SAC inactivation, APC/C^Cdc20^ activates separase but the resulting loss of (some) cohesion is accompanied by SAC reactivation and APC/C^Cdc20^ inhibition, which aborts the process of further securin degradation. Cyclin B is therefore the only APC/C^Cdc20^ substrate whose degradation at the onset of anaphase is necessary to prevent SAC reactivation. The mutual activation of tension sensitive SAC and Cdk1 creates a bistable system that ensures complete activation of separase and total downregulation of Cdk1 when all chromosomes have bioriented.

## Highlights

• Cdk1 activity is necessary for maintaining the spindle assembly checkpoint (SAC)
• SAC is reactivated during anaphase if Cdk1 activity is kept high
• APC/C blocks SAC reactivation at anaphase solely by promoting cyclin B degradation
• The SAC’s dependence on Cdk1 ensures that APC/C activation irreversible

## Graphical Abstract

**Figure:**
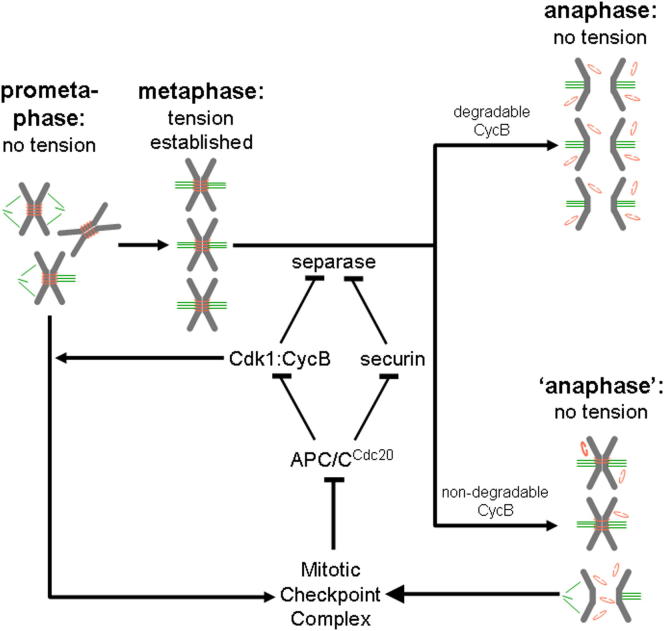

## Teaser

Rattani et al. show that Cdk1 is required for spindle assembly checkpoint (SAC) activation by unattached/tensionless chromosomes. Cdk1 inactivation through degradation of its cyclin B subunit blocks SAC reactivation during anaphase. The SAC’s dependence on Cdk1 makes separase activation irreversible.

## Results and Discussion

**Figure 1:**
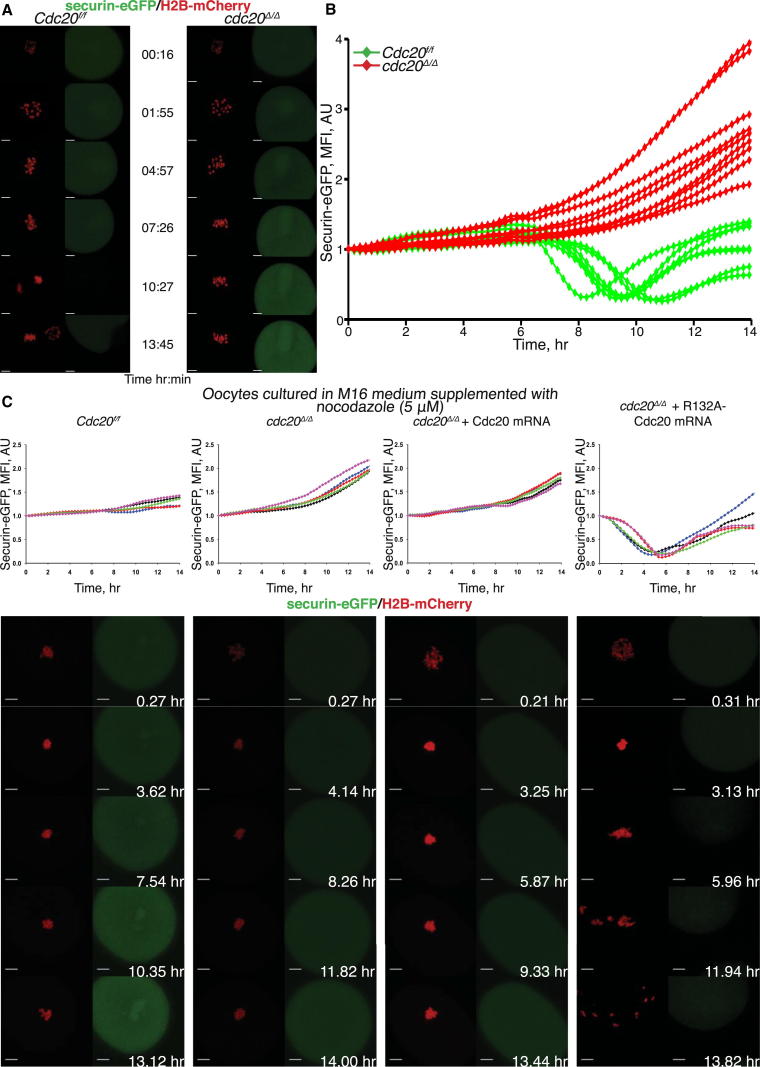
APC/C^Cdc20^ Is Responsible for Anaphase Progression in Mouse Oocytes (A) Time-lapse confocal microscopy images of oocytes expressing H2B-mCherry and securin-eGFP. Representative images from littermate control (*Cdc20*^f/f^) and *cdc20*^Δ/Δ^ oocytes are aligned to show kinetics of securin-eGFP and chromosome movements. GVBD normalized time (in hr) is indicated. Scale bars represent 10 μm. (B) Time-course measurements of securin-eGFP mean fluorescence intensity in control and *cdc20*^*Δ/Δ*^ oocytes (see also Figure S1). (C) Oocytes harvested at the GV stage from *Cdc20*^*f/f*^ and *Cdc20*^*f/f*^*ZP3-Cre* mice were microinjected with securin-eGFP, H2B-mCherry, and the indicated mRNA in M2 medium supplemented with IBMX. Oocytes were then released in IBMX-free M16 medium supplemented with nocodazole (5 μM). Chromosome movements and kinetics of securin-eGFP were visualized by time-lapse confocal microscopy. Scale bars represent 10 μm. See also Figure S1.

Disjunction of sister chromatids during anaphase is triggered by cleavage of cohesin’s kleisin subunit by separase [2]. During the early phases of mitosis and meiosis, separase is kept inactive through binding of an inhibitory chaperone called securin and phosphorylation by Cdk1:cyclin B [6, 7] and is only activated upon destruction of securin and cyclin B by a ubiquitin protein ligase called the anaphase-promoting complex/cyclosome (APC/C) [8]. The APC/C is controlled by two auxiliary proteins, Cdc20 and Cdh1, both of which are active during meiosis in mouse oocytes [9, 10]. To find out which is responsible for initiating anaphase, we generated conditional germline-specific Cdc20 knockout mice by crossing females with exon 2 of the *Cdc20* gene flanked with *LoxP* sites (*Cdc20*^f^) with males expressing Cre recombinase from the *Zona pellucida 3* promoter (*Zp3-Cre*) [11]. Live-cell imaging of oocytes injected with mRNA encoding histone-2B-mCherry and securin-eGFP revealed that securin destruction and the conversion of bivalents to dyads failed to take place in *cdc20*^Δ/Δ^ oocytes but occurred between 9 and 10 hr after germinal vesicle break down (GVBD) in *Cdc20*^f/f^ (Figures 1A and 1B; Figure S1 available online). APC/C^Cdc20^ is therefore responsible for triggering anaphase in mouse oocytes.

Securin destruction in wild-type [12] or *Cdc20*^f/f^ oocytes is blocked by nocodazole (Figure 1C). Because destruction occurred prematurely both in the presence and absence of nocodazole in *cdc20*^Δ/Δ^ oocytes injected with mRNA encoding a version of Cdc20 that cannot bind the SAC component Mad2 (R132A-Cdc20) [13, 14], but not in oocytes injected with wild-type Cdc20 mRNA (Figures 1C and S1), we conclude that SAC signaling controls the normal timing of securin degradation as well as its inhibition by nocodazole.

**Figure 2:**
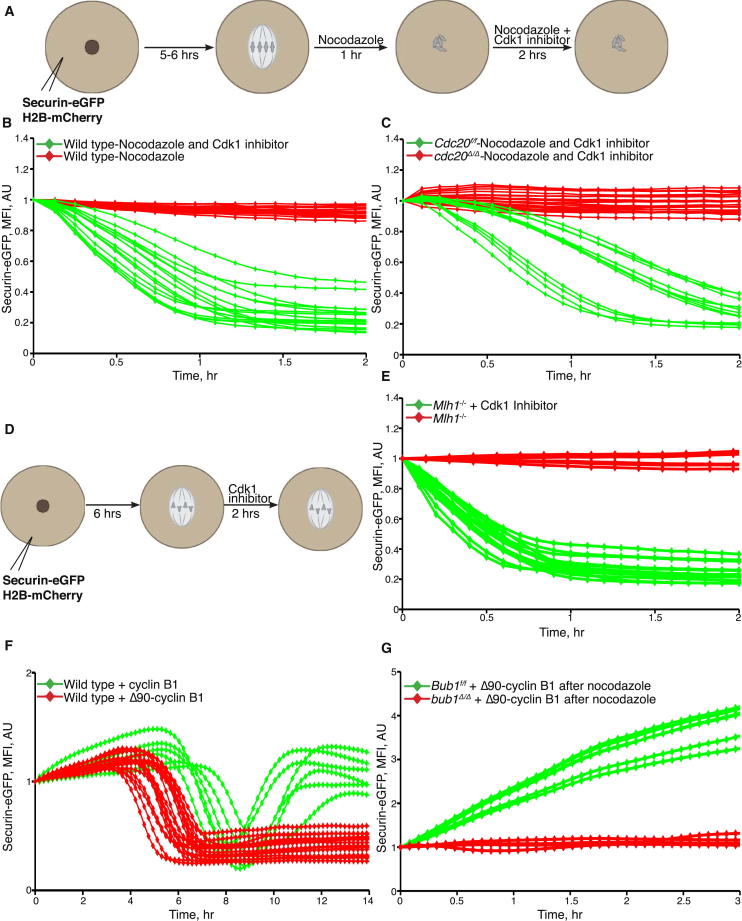
Cdk1 Is Necessary but Not Sufficient for the SAC Activity (A) Scheme of the experiment. Oocytes were microinjected with the indicated mRNAs at the GV stage. For activation of the SAC, at 5–6 hr after GVBD, corresponding to metaphase I stage, the spindle was depolymerized by 5 μM nocodazole. After 1 hr, a group of oocytes were transferred to M16 medium with nocodazole and Purvalanol A (50 μM). Chromosome movements and kinetics of securin-eGFP were visualized by time-lapse confocal microscopy. (B) Comparison of securin-eGFP mean fluorescence intensity curves from SAC-arrested oocytes cultured with (green curves) and without (red curves) Cdk1 inhibitor. (C) Time-course measurements of securin-eGFP mean fluorescence intensity after Cdk1 inhibition in *Cdc20*^f/f^ (green) and *cdc20*^Δ/Δ^ (red) oocytes cultured in M16 medium supplemented with nocodazole. (D) Scheme of the experiment. Oocytes harvested at the GV stage from *Mlh1*^−/−^ mice were microinjected with securin-eGFP and H2B-mCherry mRNA in M2 medium supplemented with IBMX. Oocytes were then released in IBMX-free M16 medium for 6 hr. At 6 hr after GVBD, a fraction of oocytes were transferred to M16 medium supplemented with 50 μM of Purvalanol A. (E) Comparison of securin-eGFP mean fluorescence intensity curves from *Mlh1*^−/−^ oocytes cultured with (green curves) and without (red curves) Cdk1 inhibitor. (F) Oocytes harvested at the GV stage were microinjected with securin-eGFP and either cyclin B1-mCherry or Δ90-cyclin B1-mCherry. The mean fluorescence intensities of the securin-eGFP signal from individual oocytes were calculated (see also Figure S2). (G) *Bub1*^f/f^ and *bub1*^Δ/Δ^ oocytes harvested at the GV stage were microinjected with securin-eGFP and Δ90-cyclin B1. Oocytes were cultured in IBMX-free M16 medium for 16 hr. At 16 hr after GVBD, 5 μM nocodazole was added to disrupt the metaphase I spindle. Kinetics of securin-eGFP was visualized by time-lapse confocal microscopy, and mean fluorescence intensities of the securin-eGFP signal from individual oocytes were calculated. See also Figure S2.

The observation that accumulation of SAC proteins at kinetochores that are not under tension is abrogated by Cdk1 inhibition raised the possibility that regulation of APC/C^Cdc20^ by the SAC depends on Cdk1 [3, 4, 15]. To test this, we treated oocytes that had recently entered metaphase with nocodazole for 1 hr, split them into two group, and treated one of these with the Cdk1 inhibitor purvalanol (50 μM) (Figure 2A). Cdk1 inhibition induced rapid securin degradation (Figure 2B). Crucially, this effect cannot be caused merely by dephosphorylation and hence activation of Cdh1 because degradation no longer took place when the experiment was repeated with *cdc20*^Δ/Δ^ oocytes (Figure 2C). Another way of eliciting an efficient SAC response in oocytes is to prevent their production of chiasmata, as occurs in oocytes lacking Mlh1 [16]. Importantly, Cdk1 inhibition also triggered rapid securin destruction in *Mlh1*^−/−^ oocytes (Figures 2D and 2E). Thus, whether triggered by loss of microtubules or by the lack of chiasmata, the SAC’s ability to inhibit APC/C^Cdc20^ in oocytes depends on Cdk1 activity.

Cdk1’s role in regulating APC/C^Cdc20^ is a complex one. On the one hand, Cdk1 inhibits APC/C^Cdc20^ by promoting the SAC, while on the other hand it has a vital role in activating APC/C^Cdc20^ in a manner that is independent of the SAC, at least in extracts from *Xenopus* eggs [17, 18]. Indeed, activation of APC/C^Cdc20^ by Cdk1 is one of the key transitions in the eukaryotic cell cycle. To see whether such an effect is also a feature of meiotic progression in mammalian oocytes in vivo, we analyzed the consequences of injecting mRNA encoding a version of cyclin B1 that cannot be degraded by APC/C^Cdc20^ (Δ90-cyclin B1) [19]. mRNA encoding nondegradable, but not wild-type, cyclin B1 advanced the onset of securin-eGFP degradation by about 2 hr (Figures 2F, S2A, and S2B). Remarkably, it had a similar effect in *bub1*^Δ/Δ^ oocytes lacking any SAC (Figure S2C). Another notable feature of oocytes expressing Δ90-cyclin B1 is that securin-eGFP fails to reaccumulate after APC/C^Cdc20^ activation. Because its synthesis persists unabated under these conditions, the lack of reaccumulation implies that once activated (in this case precociously) APC/C^Cdc20^ remains active. In wild-type oocytes or oocytes injected with wild-type cyclin B1 mRNA, securin-eGFP reaccumulates soon after its degradation, implying that APC/C^Cdc20^ is switched off soon after its activation. The fact that Δ90-cyclin B1 prevents this process implies that APC/C^Cdc20^ inactivation depends on the Cdk1 downregulation normally brought about by APC/C^Cdc20^. Both the premature activation and the maintenance of high APC/C^Cdc20^ activity by Δ90-cyclin B1 are consistent with the notion that Cdk1:cyclin B activates APC/C^Cdc20^ in a SAC-independent manner. This effect could be mediated by phosphorylation of the APC/C core by Cdk1 [17]. It might also involve inactivation of the APC/C inhibitor Emi2 [20].

Because Cdk1 activates APC/C^Cdc20^ as well as the SAC, it has both positive and negative effects on APC/C^Cdc20^ activity. To address which of these effects is dominant, we tested whether disruption of kinetochore-microtubule attachments reactivates the SAC and thereby inhibits securin degradation in the presence of high Cdk1 activity. To this end, nocodazole was added to oocytes that had activated APC/C^Cdc20^ and degraded securin in the presence of Δ90-cyclin B1. Because this induced reaccumulation of securin (indicative of APC/C^Cdc20^ inactivation) in wild-type but not *bub1*^*Δ/Δ*^ oocytes (Figure 2G), we conclude that the SAC can inactivate fully active APC/C^Cdc20^ in the presence of high Cdk1 activity. In other words, Cdk1’s activation of the SAC is dominant over its activation of APC/C^Cdc20^. Given this dominance, how then do cells normally activate APC/C^Cdc20^ in the presence of high Cdk1 activity as occurs shortly before cells undergo anaphase? The answer is that though necessary for SAC signaling, Cdk1 activity is insufficient. Also required are kinetochores that are unattached to microtubules or not under tension ([1, 21] and Figure 2G). Thus, when kinetochores come under tension and chromosomes biorient on mitotic or meiotic spindles, the SAC is turned off and Cdk1 is permitted to activate APC/C^Cdc20^ and thereby trigger its own demise as well as separase activation.

**Figure 3:**
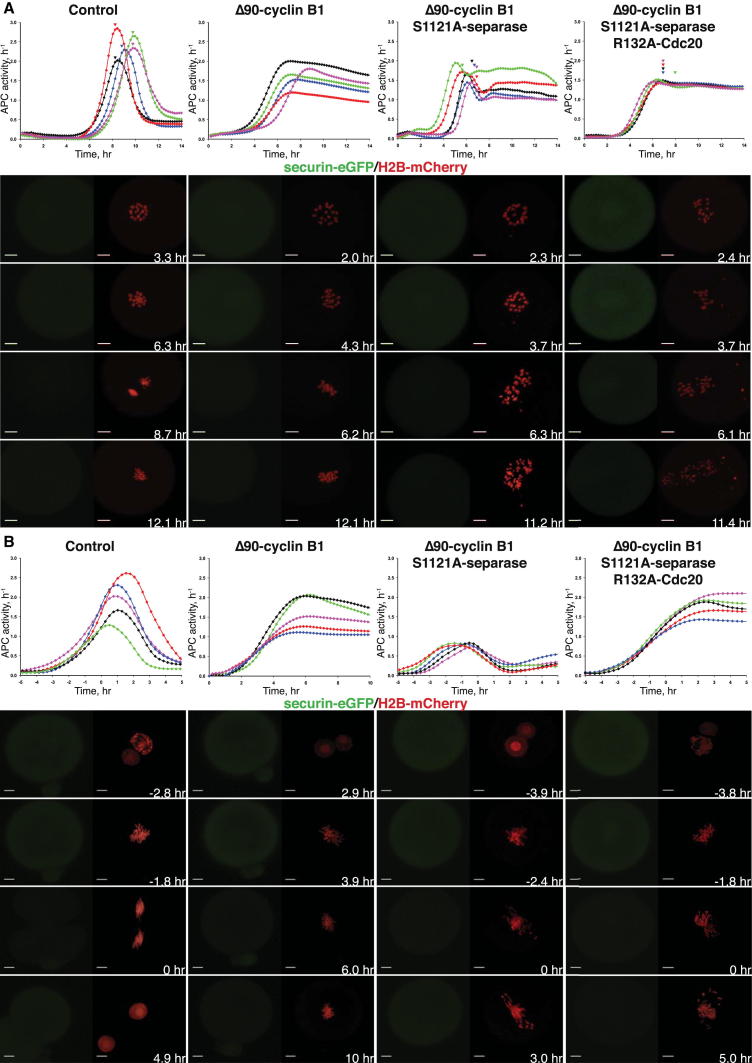
Destruction of Cyclins by the APC/C^Cdc20^ Prevents Reactivation of SAC at Anaphase (A) Oocytes harvested from wild-type control females were microinjected with securin-eGFP, H2B-mCherry, and the indicated mRNAs at the GV stage. APC/C activity, calculated using the time-course measurements of securin-eGFP mean fluorescence intensities (shown in Figure S3A), and representative time-lapsed confocal images are displayed. Scale bars represent 10 μm. (B) Pronuclear stage zygotes from wild-type mice were injected H2B-mCherry, securin-eGFP, and the indicated mRNA during interphase and imaged for 12 hr. Still images from representative movies from each group displaying securin-eGFP (left) and H2B-mCherry (right) channels are shown below the APC/C activity curves (corresponding securin-eGFP mean florescence intensities are displayed in Figure S3D). Anaphase normalized time is displayed on the x axis and on each frame. Scale bars represent 10 μm. See also Figure S3.

During a normal anaphase, separase activation by APC/C^Cdc20^ leads to cohesin cleavage, which triggers sister chromatid disjunction and segregation to opposite poles. Any loss of cohesion caused by cleavage presumably destroys the tension thought to be necessary to turn off the SAC, and yet the latter is not reactivated during anaphase and APC/C^Cdc20^ remains active for long enough to complete fully the process of disjunction. Our finding that the SAC depends on Cdk1 activity raises the possibility that the APC/C^Cdc20^ prevents this scenario because it destroys cyclin B1 and thereby downregulates Cdk1 at the same time as separase activation. If indeed cohesin cleavage destroys tension to a degree sufficient for SAC signaling and APC/C^Cdc20^ prevents this solely by destroying cyclins, then oocytes that activate separase in the presence of nondegradable cyclin B1 should reactivate the SAC during anaphase. For such an effect to be observed, it is necessary to abrogate inhibition of separase by Cdk1:cyclin B by expressing a version of separase (S1121A) refractory to Cdk1 inhibition [22]. APC/C^Cdc20^ activities were estimated by calculating the first differential of eGFP fluorescence after injection of mRNAs encoding securin-eGFP ([23] and the Experimental Procedures). In control oocytes, APC/C^Cdc20^ activity is transient, rising and then falling (Figure 3A, first column, and Figure S3A). Importantly, the decline is blocked by Δ90-cyclin B1 (Figure 3A, second column, and Figure S3B). This decline is not due to SAC reactivation, but brought rather is about by inactivation of the Cdk1 activity necessary for APC/C^Cdc20^.

Injection of mRNAs expressing S1121A separase together with Δ90-cyclin B1 led to a small transient decline in activity (Figure 3A, third column, and Figure S3A), which coincided with the chromosome segregation triggered by S1121A separase. Though modest, this decline was fully dependent on the SAC because it was abolished by coinjection of mRNAs encoding a version of Cdc20 (R132A) refractory to the SAC (Figure 3A, fourth column). These observations confirm that the SAC is indeed reactivated when tension is lost in the presence of high Cdk1 activity. However, why does this reactivation have such a modest an effect on APC/C^Cdc20^ activity? Because APC/C^Cdc20^ remains active due to the persistence of Cdk1 activity, S1121A separase also remains active, and this results in the formation of individual chromatids lacking any cohesin not dyads containing centromeric cohesin as normally occurs at meiosis I (Figures S3B and S3C). The protection of centromeric cohesion by shugoshin that normally occurs at meiosis I appears to be a transient phenomenon and persistent centromeric cohesion depends on the rapid reinactivation of separase due to reaccumulation of cyclin B and securin. Crucially, centromeric cohesin has an important role in augmenting SAC signaling during meiosis, and this phenomenon would explain why reactivation of the SAC is so weak [24].

Because a strong dependence of SAC signaling on the presence of centromeric cohesin is not a feature of the first zygotic division [24], we repeated our experiments with mouse embryos undergoing their first mitotic divisions. As in oocytes, APC/C^Cdc20^ activation in zygotes is transient (Figure 3B, first column, and Figure S3D), but expression of nondegradable cyclin B1 blocks APC/C^Cdc20^ inactivation (Figure 3B, second column). Strikingly, coinjection of mRNAs encoding S1121A separase along with Δ90-cyclin B1 not only reduced the extent of APC/C^Cdc20^ activation in the first place (i.e., its maximum), but also caused a rapid decline in activity (Figure 3B, third column). Crucially, both effects were caused by the SAC because they were abolished by coinjection of mRNAs encoding R132A-Cdc20 (Figure 3B, fourth column). Indeed, the loss of sister chromatid cohesion in the presence of high Cdk1 activity was accompanied by reaccumulation of Bub1 at kinetochores (data not shown). We conclude that in mitotic cells, where chromatids lacking cohesin are capable of generating strong SAC signals, activation of separase in the presence of high Cdk1 activity creates chromatids that then catalyze the generation of sufficient amounts of the mitotic checkpoint complex (MCC) to abort the process of APC/C^Cdc20^ full activation. The implication is that APC/C^Cdc20^ prevents its premature inactivation during anaphase by MCC complexes produced by chromatids that have lost tension by downregulating Cdk1 at the same time as activating separase. A recent work with human tissue culture cells has led to similar conclusions [25].

**Figure 4:**
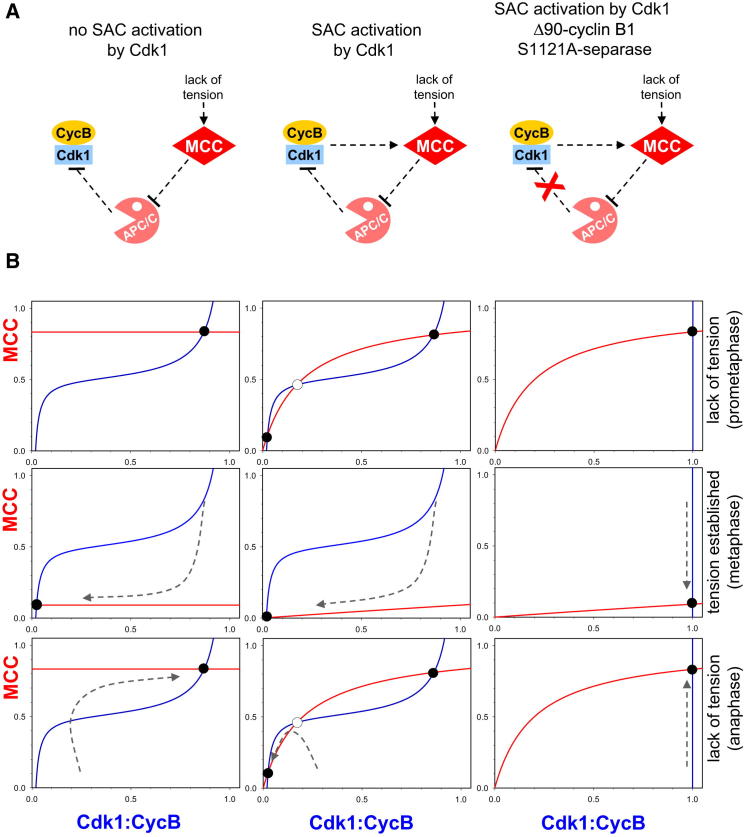
The Role Cdk1 in Irreversible Anaphase Progression (A) Influence diagram of three different model scenarios. The left panel represents the network when the SAC activation is independent of Cdk1, while the middle and right panels shows the case when SAC activation is dependent on Cdk1activity. The diagram in the right panel corresponds to the case of nondegradable cyclin B and separase (S1121A) refractory to Cdk1 inhibition. (B) The balance curves of MCC (red) and Cdk1:cyclin B (blue) are plotted for three different model scenarios (left, middle, and right) in prometaphase (top), metaphase (middle), and anaphase (bottom). Along the MCC balance curve, the rate of assembly is balanced by the rate of disassembly. The Cdk1:cyclin B balance is the locus of points where the rate of synthesis and degradation are equal.

To explore the consequences of SAC dependence on Cdk1, we created a simple mathematical model and compared its dynamical properties with and without this effect (Figure 4A). The qualitative dynamics of the system were evaluated by plotting the steady state level of cyclin B as a function of MCC and vice versa. In cells expressing wild-type degradable cyclin B, the dependence of Cdk1:cyclin B on MCC has three regimes (Figure 4B, blue curves, left and middle columns). At low MCC levels, APC/C is associated with Cdc20, which keeps cyclin B levels low. At high MCC levels, most APC/C is titrated away from Cdc20; degradation of cyclin B is therefore compromised, and Cdk1 activity is high. The transition between low and high levels of cyclin B takes place when the levels of MCC and APC/C are comparable. In cells expressing cyclin B that cannot be ubiquitylated by APC/C^Cdc20^, the level of cyclin B will be high and independent of MCC (Figure 4, right column), i.e., the blue curve is a vertical line.

The steady-state level of MCC is shown by the red curve. If the SAC is not influenced by Cdk1 activity (Figure 4, left column), the level of MCC is independent of Cdk1:cyclin B (i.e., it is a horizontal line). If Cdk1 promotes MCC formation in the presence of tensionless kinetochores, then the level of MCC increases with Cdk1:cyclin B level, but this rise is eventually limited by the amount of one of the MCC subunits. The initial slope of the curve is proportional to the number of tensionless chromosomes.

Both MCC and cyclin B are in steady state at points where the blue and red curves intersect, creating “attractors” for the system (marked by black dots). In the absence of tension, as occurs during prometaphase (Figure 4B, top three panels), the steady state is characterized by high MCC and cyclin B levels, irrespective of whether the SAC depends on Cdk1 (left versus middle panels). When all kinetochores come under tension, as occurs during metaphase, the red curves are shifted downward (Figure 4B, middle panels). In wild-type cells, the new steady state is now characterized by low MCC and cyclin B levels, a situation unaffected by whether or not the SAC depends on Cdk1. However, APC/C activation (anaphase) not only destroys cyclin B, but also activates separase, which recreates the lack of tension necessary to activate the SAC. The red curves therefore resume the properties they had during prometaphase. Under these circumstances, in cells where the SAC is independent of Cdk1 (Figure 4B, left panels), there is only a single steady state, which is characterized by high cyclin B and MCC levels. Thus, as soon as anaphase is initiated, the system will return to a prometaphase-like state associated with low APC/C and low separase activity, and anaphase will be aborted before it can be completed.

Crucially, in cells in which MCC production depends on Cdk1 (Figure 4B, middle panels), there is a second steady state (attractor) in the presence of tensionless kinetochores, an “anaphase” state characterized by low MCC and low cyclin B levels (Figure 4B, middle panels). Because cells commence anaphase with low MCC and declining cyclin B levels, the system will be attracted to this anaphase steady state and not back to the prometaphase steady state, and as a result, loss of tension caused by separase no longer reactivates the SAC. This second “anaphase” steady state is also missing in cells that express nondegradable cyclin B and S1121A separase (Figure 4, right panels), and as a consequence, loss of tension caused by APC/C activation causes the system to revert to a prometaphase-like state.

To summarize, without a dependence of the SAC on Cdk1, the anaphase control system operates as a reversible switch and orderly chromosome segregation is difficult if not impossible. Dependence of the SAC on Cdk1 enables the system to possess alternative steady states in the absence of tension; in other words, it is bistable, a property essential for the anaphase transition to be irreversible. The metaphase to anaphase transition is one of the defining moments in the life of eukaryotic cells, and the bistability unearthed by this work appears to be a fundamental aspect.

Establishing how Cdk1 promotes the SAC will be a challenge for future studies. Possible mechanisms include activation of Mps1 [26], promoting the interaction between Cdc20 and Mad2 [27], or aiding localization of the chromosomal passenger complex at kinetochores [3, 15].

Our findings emphasize that Cdk1 has multiple roles during the initiation and execution of anaphase. Cdk1-dependent phosphorylation of APC/C^Cdc20^ activates separase by promoting degradation of the anaphase inhibitors cyclin B and securin. For irreversible separase activation to be achieved, it is essential that the initial cleavage of cohesins does not lead to SAC reactivation by loss of kinetochore tension. A reactivation of SAC during anaphase progression would lead to APC/C inhibition, which would compromise full activation of separase. This potential negative feedback is not engaged during normal anaphase progression due to the dependence of SAC on Cdk1 activity. Therefore, activation of APC/C^Cdc20^ not only contributes toward the initial activation of separase, but also guarantees that its activation is switch-like and irreversible.

## Experimental Procedures

Mice were housed in the Biomedical Sciences Building at the University of Oxford, where all procedures were approved by local ethical review committee and licensed by the Home Office under the Animal (Scientific Procedures) Act 1986. Detailed experimental procedures for (1) isolation, culturing, and injection of fully grown mouse GV-stage oocytes and zygotes; (2) generation of mouse strains; (3) preparation of mRNA; and (4) live-cell imaging are described in the Supplemental Experimental Procedures. The description of mathematical model including equations and parameter values is also provided as a part of the Supplemental Experimental Procedures.

## Supplemental Information

Document S1. Supplemental Experimental Procedures and Figures S1–S3
