# Supplementary material for: Dependency of the Spindle Assembly Checkpoint on Cdk1 Renders the Anaphase Transition Irreversible
Source: Curr Biol. 2014 Mar 17;24(6):630–7. doi: 10.1016/j.cub.2014.01.033 (PMC3969274; doi:10.1016/j.cub.2014.01.033)
Supplement: Document S1. Supplemental Experimental Procedures and Figures S1–S3 [file mmc1.pdf]

Current Biology, Volume *24*

Supplemental Information

Dependency of the Spindle Assembly Checkpoint on Cdk1 Renders the Anaphase Transition Irreversible

Ahmed Rattani, P.K. Vinod, Jonathan Godwin, Kikuë Tachibana-Konwalski, Magda Wolna, Marcos Malumbres, Béla Novák, and Kim Nasmyth

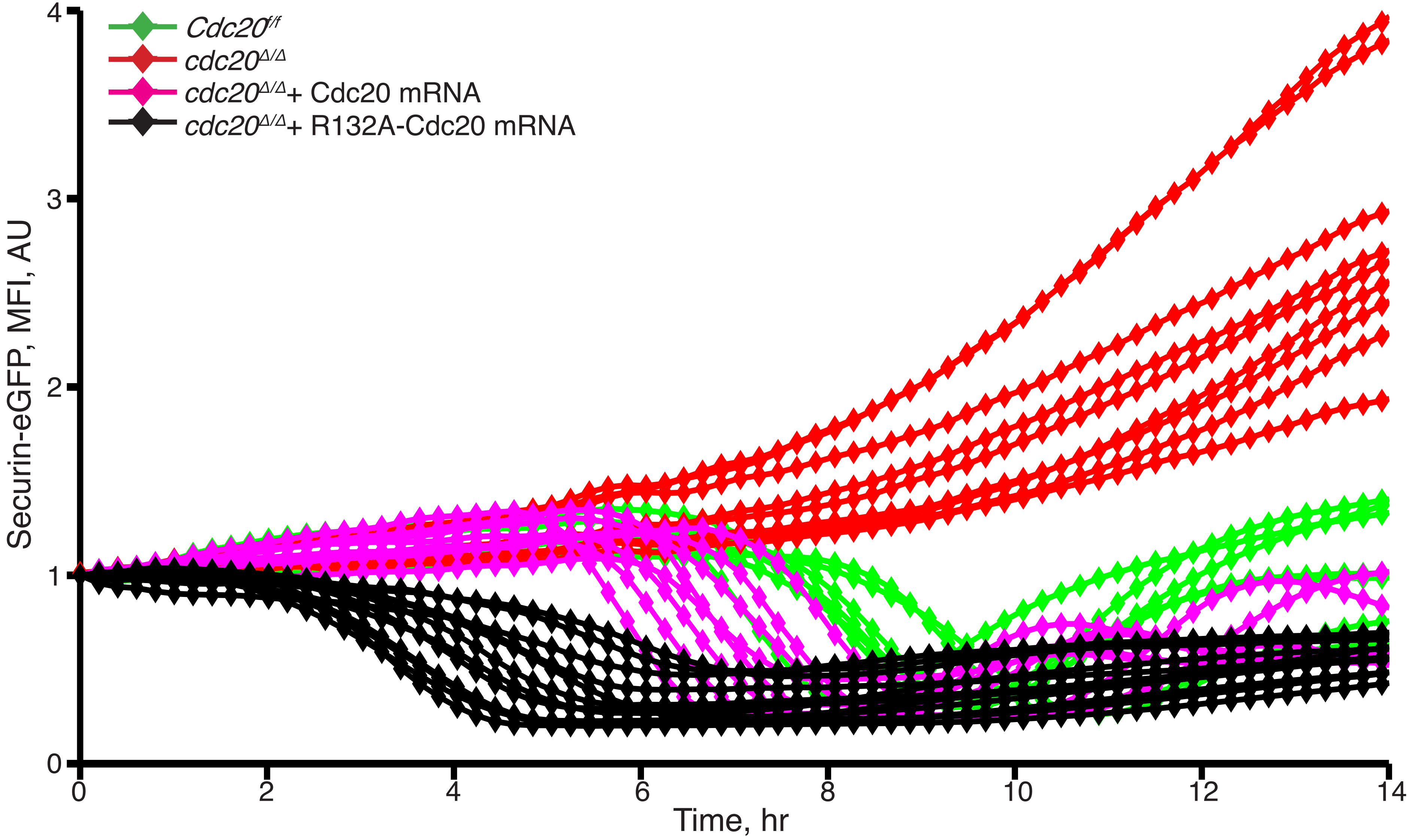
Cdc20f/f
cdc20Δ/Δ
cdc20Δ/Δ+ Cdc20 mRNA
cdc20Δ/Δ+ R132A-Cdc20 mRNA
Securin-eGFP, MFI, AU
Time, hr
0
1
2
3
4
0
2
4
6
8
10
12
14

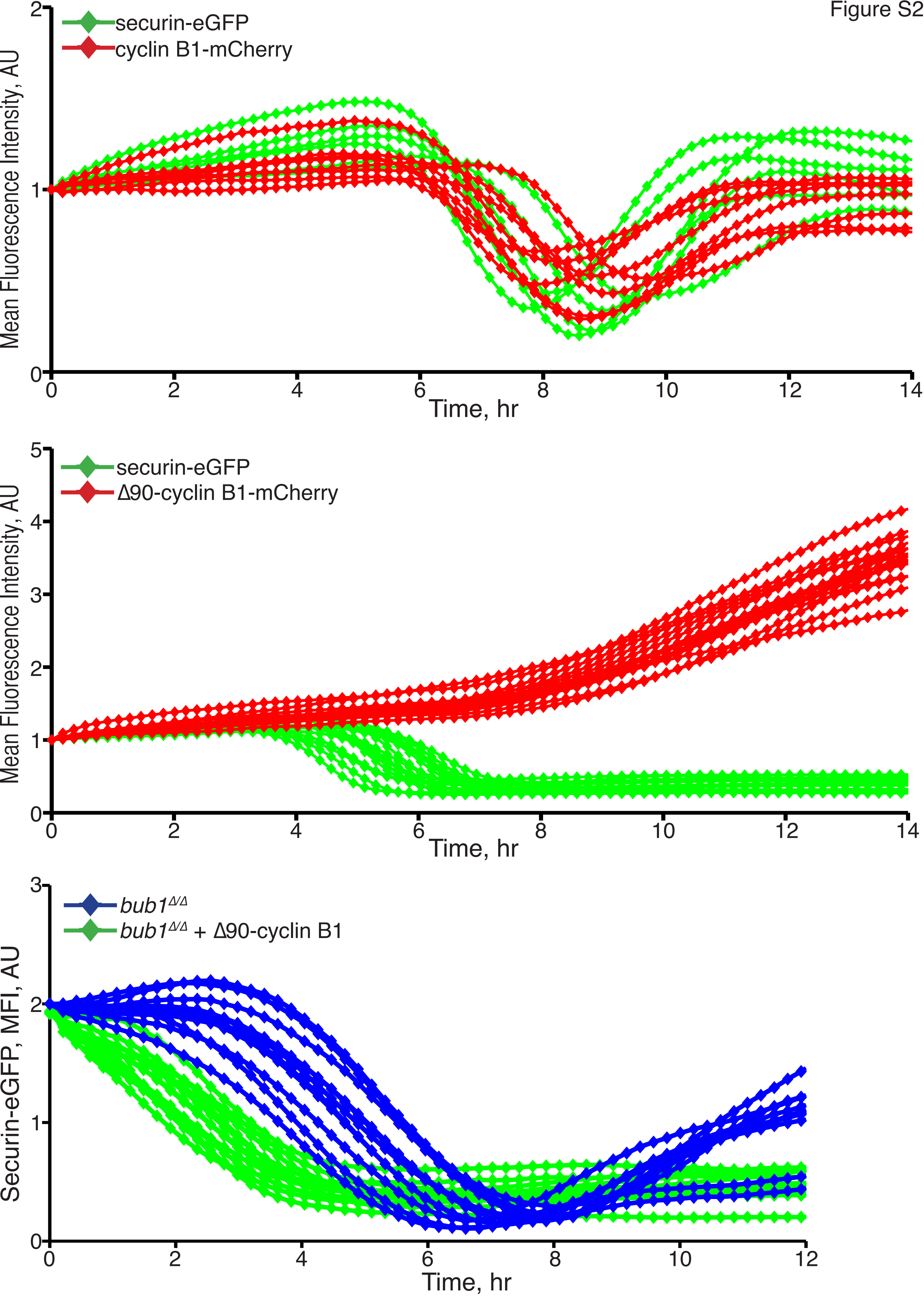

Figure S2
securin-eGFP
cyclin B1-mCherry
Mean Fluorescence Intensity, AU
2
1
0
0
2
4
6
8
10
12
14
Time, hr
securin-eGFP
Δ90-cyclin B1-mCherry
Mean Fluorescence Intensity, AU
5
4
3
2
1
0
0
2
4
6
8
10
12
14
Time, hr
bub1Δ/Δ
bub1Δ/Δ + Δ90-cyclin B1
Securin-eGFP, MFI, AU
3
2
1
0
0
2
4
6
8
10
12
Time, hr

Figure S3

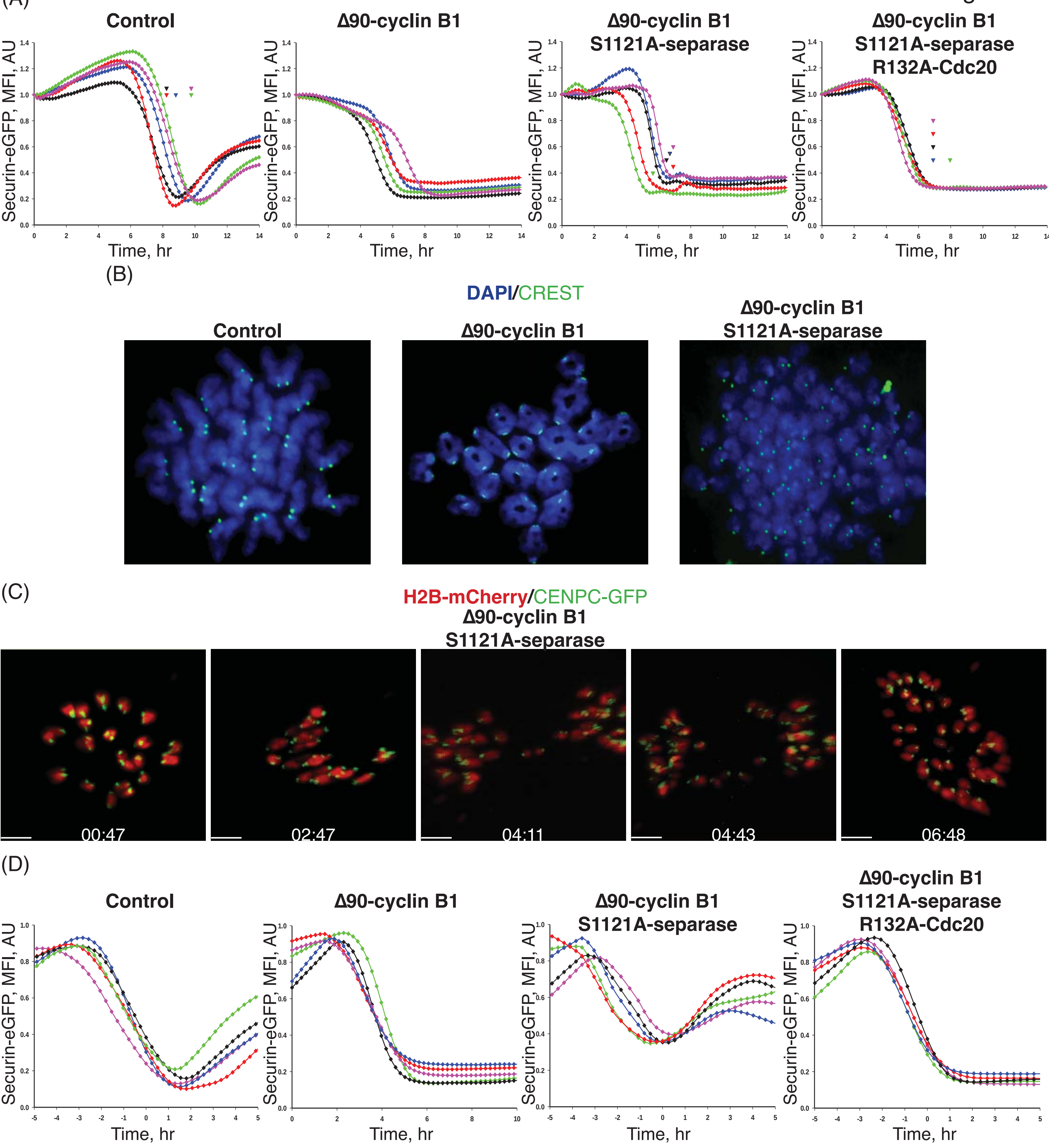
(A)
Control
Δ90-cyclin B1
Δ90-cyclin B1
S1121A-separase
Δ90-cyclin B1
S1121A-separase
R132A-Cdc20
Securin-eGFP, MFI, AU
Time, hr
(B)
DAPI/CREST
Control
Δ90-cyclin B1
Δ90-cyclin B1
S1121A-separase
(C)
H2B-mCherry/CENPC-GFP
Δ90-cyclin B1
S1121A-separase
00:47
02:47
04:11
04:43
06:48
(D)
Control
Δ90-cyclin B1
Δ90-cyclin B1
S1121A-separase
Δ90-cyclin B1
S1121A-separase
R132A-Cdc20
Securin-eGFP, MFI, AU
Time, hr

Supplemental Figure Legends

Figure S1, related to Figure 1: APC/C^{Cdc20} is required for securin destruction at metaphase to anaphase transition. Oocytes harvested at GV stage from *Cdc20$^{f/f}$* and *Cdc20$^{f/f}$ ZP3-Cre* mice were microinjected with securin-eGFP, H2B-mCherry and the indicated mRNA in M2 medium supplemented with IBMX. Time-course measurements of securin-eGFP mean florescence intensities are displayed.

Figure S2, related to Figure 2: Cyclin B1/Cdk1 activates APC/C^{Cdc20}. (A) Oocytes harvested from wild type control females were microinjected with securin-eGFP and cyclin B1-mCherry mRNAs at GV stage. Time-course measurements of mean florescence intensities are displayed. (B) Wild type control oocytes were microinjected with securin-eGFP and Δ90-cyclin B1-mCherry mRNAs at GV stage. Mean florescence intensities of GFP and mCherry signals from individual oocytes are shown. (C) *bub1$^{\Delta/\Delta}$* oocytes harvested at GV stage were microinjected with securin-eGFP and H2B-mCherry mRNAs. Fraction of oocytes received Δ90-cyclin B1 mRNA in addition to securin-eGFP and H2B-mCherry mRNAs. Time-course measurements of securin-eGFP mean florescence intensities from individual oocytes are displayed.

Figure S3, related to Figure 3: Cyclin B1 degradation prevents reengagement of SAC at anaphase. (A) Oocytes were harvested from wild type control females. Cells were microinjected with securin-eGFP, H2B-mCherry and the indicated mRNAs at GV stage. Chromosome movements and kinetics of securin-eGFP were visualized by time-lapse confocal microscopy. Time course measurements of securin-eGFP mean fluorescence intensities are plotted against GV-normalized time. (B) Oocytes microinjected at GV stage with indicated mRNAs were cultured in M16 medium for 12 h. At 12 h, chromosome spreads were prepared and slides were stained with DAPI (blue, DNA) and CREST (green, centromeres) (C) Oocytes were microinjected at GV stage with GFP-CENPC, H2B-mCherry, Δ90-cyclin B1-mCherry and S1121A-separase mRNAs. Time-lapse confocal images show that centromeric cohesion was cleaved in oocytes injected with Δ90-cyclin B1-mCherry and S1121A-separase. (D) Pronuclear stage zygotes from wild type mice were microinjected H2B-mCherry, securin-eGFP and the indicated mRNA during interphase and imaged for 12 h. Time course measurements of securin-eGFP mean fluorescence intensity are plotted against anaphase-normalized time.

Supplemental Experimental Procedures

Generation of Mouse Strains

Generation of the *Cdc20* targeted mice has been described [S1]. Cdc20 protein was depleted specifically in mouse oocytes with a Zp3-Cre transgene to delete exon 2 from a floxed *Cdc20* allele.

Isolation and Culture of Cells

Fully grown mouse GV oocytes were harvested from ovaries excised from 6-12 week old female mice. Mature follicles were isolated using sterile insulin needles in M2 medium (Sigma Aldrich) supplemented with 200µM IBMX (Sigma Aldrich). Following microinjection of in-vitro transcribed mRNA in droplets of M2 medium with IBMX, oocytes were transferred to IBMX-free M16 medium at 37°C and 5% CO_2 for time-lapse confocal imaging.

For zygotes isolation, 3- to 4 week-old females were injected intraperitoneally with 5 IU of PMSG and 48 h later with 5 IU of hCG. Females were paired with B6CBAF1 (Harlan) male mice and zygotes were retrieved from oviductal ampullae at 17–20 h post-hCG. Cumulus-enclosed zygotes were washed through M2 medium (Sigma Aldrich), and cultured in M16 in the presence of 5% CO_2 at 37°C.

Preparation of mRNAs

Capped mRNA constructs with a poly-A tail were transcribed using T3 or T7 Ultra mMESSAGE kit (Ambion) from plasmid cDNA encoding H2B-mCherry, securin-eGFP, cyclin B1-mCherry, Δ90-cyclin B1 mCherry, Δ90-cyclin B1 (untagged), R132A mutant of Cdc20 and S1121A mutant of separase. Wild type Cdc20 and separase were mutated by site-directed mutagenesis.

Microinjection of mRNA

GV arrested oocytes were injected with 5-10 pl mRNA at a final concentration of 0.1 mg/ml prepared in RNase-free water (Ambion) using a Pneumatic PicoPump (World Precision Instruments). Following microinjection of mRNA, oocytes were cultured for 1-2 h in M16 medium supplemented with IBMX. Oocytes were then washed in inhibitor-free M16 medium and thereafter cultured at 37°C and 5%CO_2.

Live Cell Confocal Microscopy

For time-lapse imaging experiments, oocytes were cultured in a PeCon environmental microscope incubator at 37°C and 5% CO_2. A Zeiss LSM510 META confocal microscope equipped with PC-Apochromat 63x/1.2 NA water immersion and PC-Apochromat 20x/0.8 NA objective lenses was used for image acquisition. 488-nm and 561-nm excitation wavelengths and BP 505-550 and LP 575 filters were used to detect GFP and mCherry, respectively. During live-cell imaging, chromosomes labelled with H2B-mCherry were tracked with an EMBL-developed tracking macro[S2]. Image stacks of 8 to 16 slices of 1.5-2.5 µm were captured every 10-15 mins for 12-14 h.

Chromosome Spreads

Chromosome spreads from mouse oocytes were prepared as previously described [S3, S4]. CREST (1:250; Davis Lab) antiserum was used to mark kinetochores.

Calculation of APC/C activity from securin levels

The temporal pattern of APC/C activity during meiosis was calculated from the securin time-course data (S) and its first time-derivative (dS/dt) using the formula derived in [S5]:

$$APC = \frac{k_{synth} - dS/dt}{S} - k_{degr}$$

where k_{synth} and k_{degr} represent the rate constants ($time^{-1}$) for securin synthesis and APC/C independent degradation, respectively.

Mathematical modelling

The antagonism between Cdk1:cyclin B and MCC (an effector of SAC) on Fig. 4 was described by the following differential and algebraic equations based on law of mass action kinetics. cyclin B ($CycB_T$) synthesis is constant and its degradation is APC/C (APC) dependent. MCC is activated proportional to unattached kinetochores (uKT) and active cyclin B (CycB). MCC_t refers to the sum of free and APC/C bound MCC. Active (not MCC bound) APC/C (APC) is calculated by assuming reversible, equilibrium binding with K_{diss} dissociation constant. Cdk1 activity (Cdk1) is calculated by expressing the Cdk1 inhibitor concentration relative to its IC_{50} value. The two differential equations used to describe the dynamics of Cdk1:cyclin B and the Mitotic Checkpoint Complex (MCC) make the following assumptions. 1), The rate of cyclin B synthesis is constant, while its degradation is controlled by APC/C^{Cdc20} dependent ubiquitylation. 2), Tensionless kinetochores promote assembly of the Mitotic Checkpoint Complex (MCC) in a Cdk1 activity dependent or independent manner. 3), The rapid and reversible binding of MCC within the APC/C blocks association of APC/C with Cdc20 and thereby inhibits cyclin B degradation. Cdc20 is only implicit in the model, because we assume that its level is not limiting, as demonstrated by data presented in this paper.

$$\frac{d\,CycB_T}{dt} = k_s - (k_d' + k_d \cdot APC) \cdot CycB_T$$

$$\frac{d\,MCC_t}{dt} = k_a \cdot uKT \cdot Cdk1 \cdot (Mad2_T - MCC_t) - k_i \cdot MCC_t$$

$$APC = \frac{APC_T - MCC_t - K_{diss} + \sqrt{(APC_T - MCC_t - K_{diss})^2 + 4\,APC_T\,K_{diss}}}{2}$$

$$Cdk1 = \frac{CycB_T}{1 + Inh}$$

Parameter values: $k_s = 0.01$, $k_d' = 0.01$, $k_d = 1$, $k_a = 5$, $k_i = 1$, $K_{diss} = 0.001$, Inh=0. The APC/C dependent degradation rate constant for cyclin B (k_d) is set to zero for non-degradable cyclin B (Fig. 4, right column). uKT equals to 1 in the absence of tension (prometaphase and anaphase, Fig. 4B top and bottom rows) and it is set to a small number (0.02) when tension is

established (metaphase, Fig. 4B middle row). The MCC balance curve on Fig. 4B left column was calculated by removing Cdk1 from the activation term of MCC_t differential equation. Relative protein levels: $Mad2_T = 1$, $APC_T=0.5$

Supplemental References

S1. Manchado, E., Guillamot, M., de Carcer, G., Eguren, M., Trickey, M., Garcia-Higuera, I., Moreno, S., Yamano, H., Canamero, M., and Malumbres, M. (2010). Targeting mitotic exit leads to tumor regression in vivo: Modulation by Cdk1, Mastl, and the PP2A/B55alpha,delta phosphatase. Cancer Cell *18*, 641-654.

S2. Rabut, G., and Ellenberg, J. (2004). Automatic real-time three-dimensional cell tracking by fluorescence microscopy. J Microsc *216*, 131-137.

S3. Hodges, C.A., and Hunt, P.A. (2002). Simultaneous analysis of chromosomes and chromosome-associated proteins in mammalian oocytes and embryos. Chromosoma *111*, 165-169.

S4. Peters, A.H., Plug, A.W., van Vugt, M.J., and de Boer, P. (1997). A drying-down technique for the spreading of mammalian meiocytes from the male and female germline. Chromosome Res *5*, 66-68.

S5. McGuinness, B.E., Anger, M., Kouznetsova, A., Gil-Bernabe, A.M., Helmhart, W., Kudo, N.R., Wuensche, A., Taylor, S., Hoog, C., Novak, B., et al. (2009). Regulation of APC/C activity in oocytes by a Bub1-dependent spindle assembly checkpoint. Curr Biol *19*, 369-380.
